# Simulated-use validation of a sponge ATP method for determining the adequacy of manual cleaning of endoscope channels

**DOI:** 10.1186/s13104-016-2066-7

**Published:** 2016-05-04

**Authors:** Michelle J. Alfa, Nancy Olson

**Affiliations:** St. Boniface Research Centre, 351 Tache Ave, Winnipeg, MB R2H 2A6 Canada; Department of Medical Microbiology, University of Manitoba, Winnipeg, MB Canada

**Keywords:** Sponge-sample, Cleaning adequacy, Cut-off

## Abstract

**Background:**

The objective of this study was to validate the relative light unit (RLU) cut-off of adequate cleaning of flexible colonoscopes for an ATP (adenosine tri-phosphate) test kit that used a sponge channel collection method.

**Methods:**

This was a simulated-use study. The instrument channel segment of a flexible colonoscope was soiled with ATS (artificial test soil) containing approximately 8 Log_10_*Enterococcus faecalis* and *Pseudomonas aeruginosa*/mL. Full cleaning, partial cleaning and no cleaning were evaluated for ATP, protein and bacterial residuals. Channel samples were collected using a sponge device to assess residual RLUs. Parallel colonoscopes inoculated and cleaned in the same manner were sampled using the flush method to quantitatively assess protein and bacterial residuals. The protein and viable count benchmarks for adequate cleaning were <6.4 ug/cm^2^ and <4 Log_10_ cfu/cm^2^.

**Results:**

The negative controls for the instrument channel, over the course of the study remained low with on average 14 RLUs, 0.04 ug/cm^2^ protein and 0.025 Log_10_ cfu/cm^2^. Partial cleaning resulted in an average of 6601 RLUs, 3.99 ug/cm^2^, 5.25 Log_10_ cfu/cm^2^*E. faecalis* and 4.48 Log_10_ cfu/cm^2^*P. aeruginosa*. After full cleaning, the average RLU was 29 (range 7–71 RLUs) and the average protein, *E. faecalis* and *P. aeruginosa* residuals were 0.23 ug/cm^2^, 0.79 and 1.61 Log_10_ cfu/cm^2^, respectively.

**Conclusions:**

The validated cut-off for acceptable manual cleaning was set at ≤100 RLUs for the sponge collected channel ATP test kit.

## Background

Cleaning of flexible endoscopes is still predominately a manual process that is fraught with errors [1–4]. As reviewed by Ofstead et al. [5], human factors play a significant role in the efficacy of the manual cleaning of flexible endoscopes and only 1.4 % of all flexible GI endoscopes reprocessed that they evaluated had all the steps properly performed. The recent outbreaks of carbapenem-resistant enterobacteriaceae (CRE) associated with improperly reprocessed flexible duodenoscopes [1, 6] has led to questions regarding how endoscopy clinics can ensure their endoscope reprocessing is adequate. Culture of fully reprocessed ERCP duodenoscopes has been suggested as a means of detection of specific pathogens such as CRE [6], however, this would require quarantine of the endoscopes until the culture results are back (usually 48 h). Culture to determine if ERCP endoscopes harbor CRE is an excellent initial assessment. However, there needs to be a quality system approach to endoscope reprocessing to ensure on an ongoing basis that the basic reprocessing steps are being performed properly.

Recently, the development of rapid audit tools (e.g. detection of residual organic residues [7–9], or adenosine tri-phosphate (ATP) residues [10–14]) has allowed users to assess the efficacy of their manual cleaning such that improperly cleaned flexible endoscope channels can be re-cleaned prior to going to the high level disinfection (HLD) step. This process of rapidly auditing the cleaning and repeating it, if needed, ensures that an inadequately cleaned endoscope will not be used on a subsequent patient. This reduces the risk of improperly reprocessed flexible endoscopes being responsible for transmission of infectious diseases. Testing for residual ATP is one of the most widely published methods to rapidly audit cleaning compliance of flexible endoscopes [8, 10, 11, 13–15].

There have been a number of publications related to using ATP to monitor channel cleaning where the sample methods consists of a flush or flush-brush-flush of the channel [9–11, 13–15]. Since the sensitivity of different manufacturer’s ATP test kits varies [12], it is crucial that each manufacturer validate the “cut-off” level of ATP for adequate cleaning for their specific test kit. There have been reports validating the cut-off for some ATP test kits [13], but there are no published reports of validation of an ATP test kit that uses a “sponge” sample collection method to assess endoscope channel cleaning.

The objective of this study was to assess the efficacy of sample collection using a moistened sponge passed through the colonoscope instrument channel and to determine the appropriate RLU cut-off for this test kit that correlates with adequate cleaning as defined by protein and bacterial markers.

## Methods

No research or ethics approval was needed, as this was a totally in vitro study.

### Flexible endoscopes

Two Fujinon colonoscopes Model EC-530HL (Fujinon, Saitama City, Japan) were used for this study (colonoscope #1 and colonoscope #2). These colonoscopes were provided by Ruhof for the purposes of this study and were returned to Ruhof at the completion of the study. Colonoscope #1 and colonoscope #2 were alternated during the testing so they were used equally throughout the experimental testing protocol. Only instrument channel testing was performed (i.e. soiling and sample collection were only done for the instrument channel segment). After each experiment all channels and all surfaces of the colonoscope were cleaned and then the colonoscope was sterilized as per the endoscope manufacturer’s instructions. The STERIS System 1 (STERIS Corp., Mentor, OH) was used for sterilization of the colonoscopes (peracetic acid was the sterilant). Post-sterilization, all colonoscopes channels were flushed with 70 % ethanol and forced air was used to dry all channels prior to storage.

### Colonoscope cleaning methods tested

#### Full-cleaning

Leak testing was performed using a LT-7 hand-held leak tester (Fujinon) while the colonoscope was fully immersed. Full cleaning was performed as per the colonoscope manufacturer’s instructions using Ruhof Endozime^®^ Bio-Clean enzymatic detergent (Ruhof, Mineola, New York, USA) at a use-dilution of 6 mL/L and a total contact time of 2 min at room temperature. While fully immersed, the colonoscope was wiped with a lint-free cloth, the outlets of the instrument channel port and the air–water port were brushed with short brushes (Ruhof) and all channels were brushed three times with a double-ended channel cleaning brush (Ruhof). Subsequently, all channels were flushed with detergent manually using the CA-510 cleaning adaptor for G5 series Fujinon colonoscopes. A total of 90 mLs of detergent was flushed through each channel. The colonoscope was then transferred and immersed in a basin of tap water, the exterior wiped with a lint-free cloth and a total of 90 mLs of water was flushed through each channel using the CA-510 adaptor.

#### Partial cleaning

The partial clean consisted of a 50 mL flush of sterile reverse osmosis (sRO) water through the instrument channel segment (no brushing of this channel).

#### Microorganisms

*Enterococcus faecalis ATCC* 29212 and *Pseudomonas aeruginosa ATCC* 27853 were stored at −70 °C as stock cultures and were sub-cultured three times on Tryptic Soy agar containing 5 % (v/v) sterile sheep blood (BA) prior to being used in experimental protocols. This consecutive 3 day sub-culturing protocol was used to ensure the bacteria were actively replicating prior to being used in the experimental protocol.

#### Test soil

For soiling the colonoscope channel, the organic challenge consisted of ATS (artificial test soil) containing ~10^8^ cfu/mL of *Enterococcus faecalis ATCC* 29212 and *Pseudomonas aeruginosa ATCC* 27853. The ATS was freshly prepared and does contain ATP (derived from serum and blood in the test soil), blood, serum and thickening agents (US Patent No. 6447990). The ATS used for this study contained 1,390,000 RLUs/mL (average of five replicates).

#### Colonoscope inoculation

The ATS containing *E. faecalis* and *P. aeruginosa* was drawn up into a 30cc syringe that was attached to the instrument channel port on the colonoscope. The test soil was flushed through the instrument channel segment and excess soil flushed out with air. The inoculated channel was allowed to dry passively for 1 h. After drying, a 30cc syringe containing 30 mL sRO water was attached to the instrument channel port and flushed through the instrument channel segment (similar to a bedside flush used during patient-procedures). This pre-rinse was done to reduce the channel soiling to ensure the RLU level did not exceed 9999 since the Ruhof luminometer has a maximum RLU reading of 9999 (i.e. any test sample with RLUs ≥9999 will all give the same reading).

#### Sample harvesting of endoscope channel

The ATP test kit manufacturer’s instructions indicated that once the sponge has been passed through the endoscope channel, the entire sponge should be cut off and placed into the ATP test device. This test method would not allow quantitation of protein or viable count from that sponge sample. As such we developed a sponge elution method so that ATP, protein quantitation and viable count of the sponge sample could be determined. The three sample methods included: Sponge sample (as described by the manufacturer of this ATP test kit), sponge elution sample (done for research purposes only) and the fluid flush sample (done for research purposes to determine the protein and viable count in the colonoscope channel).

#### Sponge sample (ATP test only)

The Ruhof ATP test swab (ATP Complete), the Ruhof Test Instrusponge™ Channel Testing Sponge and the Ruhof ATP Complete hand-held chemiluminometer (Ruhof) were used for this study. Collection of the sample from a colonoscope channel consisted of passing the Instrusponge once through the instrument channel segment. The sponge was then cut off and placed into the ATP Test tube, which was activated and then placed into the hand-held ATP luminometer, and the RLUs determined as per the manufacturer’s instructions.

#### Sponge elution sample (ATP, protein, viable count testing)

The ATP test kit evaluated in this study uses a sponge to collect channel samples. Instead of bristles on the channel brush there is an adsorbent sponge that fits into the channel snuggly thereby creating friction against the inner channel surface. We wanted to determine at what RLU cutoff does the ATP test have protein and viable counts that meet the benchmarks for adequate cleaning. Since the sponge sample method was intended only to be used for ATP determination we evaluated this elution method to determine if ATP, protein and viable count could be reliably determined from the sponge eluate (i.e. could all the test parameters we wanted to compare be done on one sponge sample?). The sponge provided in the ATP test kit was moistened with sRO water and passed through the instrument channel segment. Once the sponge emerged from the distal tip, it was aseptically cut off and placed in 20 mL of sRO water. The sample was eluted by mixing well using a vortex mixer. The eluted fluid was used for viable count, ATP and protein determinations. For ATP measurement of the sponge elution sample, the sponge in the eluted sample was aseptically removed and excess fluid allowed to drip off the sponge, which was then transferred into the ATP test tube and then tested for ATP, as described for the ATP test sponge sample.

#### Flush sample (ATP, protein viable count testing)

A 30cc sterile syringe containing 20 mL sRO water was attached to the instrument channel port and the fluid flushed through the instrument channel segment and collected in a sterile container. This fluid was used for viable count, ATP and protein determinations. For the ATP measurement of the flush sample, the sponge was inserted into the sample and allowed to absorb fluid (each sponge absorbs 0.041 mLs of fluid). The ATP sponge was then cut off and transferred into the ATP test tube and then tested for ATP as described for the ATP test sponge sample.

### Assay test methods

For each testing protocol there were between 3–5 replicates performed (i.e. N = 3 or N = 5 where samples were collected from sequential testing using colonoscope #1 and colonoscope #2). Table 1 summarizes the testing protocol for each type of endoscope sample collected.

**Table 1:** Tests performed on each colonoscope channel sample type

Test method	Instrument channel segment^a^		
	Samples collected (N = 5)		
	Flush	Sponge elution	Sponge
ATP	X	X	X
Protein quantitation	X	X	Not done
Viable count	X	X	Not done

^a^Each sample is from a different colonoscope

#### Viable bacterial count (for flush or sponge elution fluid samples)

Quantitation of viable bacteria was performed using standard serial 1:10 dilutions where 0.1 mLs of each dilution was spread over the surface of a BBL™CHROMagar™ Orientation media plate (Becton Dickenson, Orville, ON, Canada). The limit of detection for the viable count assay was 10 cfu/mL.

#### Protein assay (for flush or sponge elution fluid samples)

The samples collected were assayed for protein using the QuantiPro BCA assay kit, which includes an internal bovine serum albumin protein standard, and is a quantitative assay based on bicinchoninic acid (Sigma, St Louis, Missouri). The protein assay was performed as per the manufacturer’s instructions and had a limit of detection of 0.5 μg/mL.

#### Benchmarks for adequate manual cleaning

The manual cleaning benchmarks for flexible endoscope channels that were established by Alfa et al. [7] and supported by Pineau et al. [8] were used. If manual cleaning of the endoscope channel has been adequate then there should be ≤6.4 µg/cm^2^ of protein and ≤4 Log_10_ cfu/cm^2^ of bioburden.

## Results

The data in Table 2 show that the protein residuals collected on the sponge are not well eluted as the μg/cm^2^ for protein collected by the sponge was much lower than that collected using a channel flush method. Also the residual ATP detected in the channel by the direct sponge method was higher than the RLU levels detected by the flush method. Based on these findings, the remainder of the simulated-use testing focused on using parallel soiled colonoscopes where the protein and viable count residuals post-treatment were determined using the flush collection method and the ATP was determined using the direct sponge collection method on a second endoscope channel inoculated at the same time.

**Table 2:** Comparison of residual ATP detected in a soiled colonoscope instrument channel evaluated using various sample collection methods

Channel sample collection method^a^	RLUs^b^ (standard deviation)	Protein μg/cm^2^ (standard deviation)
Sponge direct^c^	9304.2 (1028.6)	Not applicable
Sponge eluted (20 mL sample)	2511.4 (1142.1)	0.54 (0.14)
Flush only (20 mL sample)	2850.4 (1261.6)	26.66 (5.98)

^a^Data for each test parameter represents the average of five replicate endoscopes tested (N = 5)

^b^ATP was reported as relative light units (RLUs)

^c^The range of RLUS for the sponge direct collection method was 7709–9999 with three of the five replicates having RLU values of 9999 (i.e. maximum RLU value read by the luminometer). This 9999 maximum RLU value was used for the purposes of calculating the average and standard deviation recognizing that it was not an exact RLU value

To validate the appropriate RLU cutoff of this sponge ATP channel collection method, parallel soiling of the colonoscope instrument channel segment was used (Table 3). The negative controls from fully reprocessed colonoscope instrument channels (i.e. post-cleaning and sterilization) ranged from 1 to 39 RLUs over the course of the study. This indicated that even with repeated ATS soiling, cleaning and sterilization that the level of RLUs in the instrument channel returned to similar low values after full reprocessing was performed. This negative control data was captured and presented separately (i.e. not subtracted from the positive, partial clean or full-clean data) to ensure the data for “full cleaning” was not affected by repeated round of soiling and testing. After full manual cleaning of the colonoscope channel, the ATP level detected by the sponge method was on average 29.0 ± 36.39 RLUs (range 7–71 RLUs).

**Table 3:** Comparison of residual ATP detected by the direct sponge method versus level of residual protein and viable count in colonoscope instrument channels

	Sponge sample^b^ (N = 3)	Flush sample^c^ (N = 3)		
Test parameter^a^	RLUs (Std)	Protein μg/cm^2^ (Std)	*E. faecalis* Log_10_ cfu/cm^2^ (Std)	*P. aeruginosa* Log_10_ cfu/cm^2^ (Std)
Positive control Soiled but no cleaning	9747.00 (436.48)	335.4 (65.55)	6.782 (0.212)	6.669 (0.192)
Partial clean Soiled, 50 mL water flush and no brushing	6601.00 (1684.00)	3.99 (3.16)	5.254 (0.142)	4.476 (0.253)
Full cleaning Soiled, cleaned pre-HLD	29.00 (36.39)	0.23 (0.07)	0.787 (0.364)	1.608 (0.269)
Negative control Soiled, cleaned post-HLD and storage	14.00 (21.66)	0.04 (0.07)	0.025 any organism (0.090)	

^a^Data for each test parameter represents the average and standard deviation (Std) of three replicate endoscope tests (N = 3)

^b^ATP was measured as relative light units (RLUs)

^c^The protein and viable counts were done on a parallel set of colonoscopes that were soiled in the same manner and were harvested using the flush method of sample collection

## Discussion

Most traditional ATP test kits use swabs to sample surfaces and use fluid to collect samples from endoscope channels [7, 9, 10, 13–15]. Our report is the first to experimentally validate an appropriate RLU cut-off for the sponge-based ATP test kit. We recommend a cutoff of 100 RLUs for this sponge-based ATP test kit. Although the cut-off could have been set at 71 RLUs, as this was highest RLU found post-cleaning, we believe that there needs to be some margin allowed for variability in the ATP assay. The 100 RLU cut-off ensures that the maximum RLU level detected post cleaning during simulated-use testing was well below this cutoff. Furthermore, this cutoff represented two standard deviations higher than the mean RLU providing a reasonable margin to accommodate the variability in low RLU levels post-cleaning. Table 3 data indicated that a cut-off of ≤100 RLUs for adequate cleaning would ensure that the protein and bioburden levels in this channel would be well below the established cut-off of ≤6.4 µg/cm^2^ protein and ≤4 Log_10_ cfu/cm^2^ bioburden.

The 100 RLU cut-off for this sponge channel-sample method is different from the 200 RLU cut-off established for the flush channel-sample method previously reported by our lab for a different ATP monitoring test kit [13, 14]. This likely reflects differences in both the channel sample method and the hand-held ATP detection luminometer parameters. Differences in sensitivity and luminometer characteristics of different manufacturer’s of ATP detection kits have been clearly outlined by Sciortino et al. [12] and Aiken et al. [10]. Data from the current study and from our previously published report [13] confirm the importance of each manufacturer clearly validating the appropriate “cut-off” for clean that relates to the specific adaptation of their ATP test kit (i.e. the cut-off for “clean” may be different for the same test kit for different applications such as environmental monitoring versus monitoring of manual cleaning for flexible endoscope channels and it may also be different for kits manufactured by different companies).

We did evaluate a similar sponge collection method previously [13] however, the ATP test in that evaluation was done only on the eluted sponge. Our current study data indicated that the direct ATP sponge collection method “concentrates” the residual material collected from the channel (i.e. is not diluted by eluting in fluid) thereby ensuring the highest detectable ATP signal. The only disadvantage is that other markers for cleaning adequacy cannot be assessed on the same sponge that was used for ATP testing.

Our simulated-use negative control data do confirm that for colonoscopes the level of ATP in the instrument channel was returned to a reproducibly low level for all fully reprocessed colonoscopes for the two different manufacturers ATP test kits that we have evaluated. For the flush only elution method used for Alfa et al. [13], the ATP level for the instrument channel of fully reprocessed colonoscopes was 25.5 RLUs (standard deviation of 21.4 RLUs) compared to 14 RLUs (standard deviation of 21.7 RLUs) for the ATP sponge collection method used in the current study.

It should be noted that ATP test methods are excellent for monitoring manual cleaning adequacy for flexible GI endoscopes, however this test cannot replace culture for detection of specific pathogens present in low levels post-HLD. It requires from 10^2^–10^3^ cfu of bacteria per sponge sample to detect 1 RLU (i.e. 10^4^–10^5^ cfu are needed to detect 100 RLU). Furthermore, if ATP was detected post-disinfection/sterilization it would not be possible to differentiate whether the ATP residuals were from bacteria or from other organic residuals such as patient-secretions. Sites that want to assess their ERCP endoscopes for carbapenem-resistant Enterobacteriaceae or other specific types of bacteria will need to perform culture. Further studies are needed to determine whether the ATP test methods could detect biofilm accumulation within endoscope channels.

A limitation of this study was that we did not have the history of the two colonoscopes used for our simulated-use studies so do not know if their usage history was similar. In addition, only the instrument channel segment of the suction channel from colonoscopes was evaluated and no other channels or endoscope types were assessed. Currently there are no sponges that fit into the other smaller channels nor could this method be used for the un-sealed elevator guide-wire channel as the sponge could not fit into this channel. For the instrument channels of gastrointestinal (GI) flexible endoscopes that have similar internal channel diameters (e.g. 3.7–4.0 mm diameter) we would expect the sponge to provide similar sample collection efficacy from the channel surface results regardless of the endoscope type (i.e. colonoscope versus duodenoscope versus gastroscope). In addition, only the one specific type of channel sponge was evaluated. Despite these limitations, the sponge channel collection method provides an easy to perform, rapid and sensitive method for routine monitoring of the cleaning adequacy for flexible GI endoscopes. The frequency of testing the adequacy of manual cleaning (i.e. every endoscope every time versus a portion of endoscopes tested per week) still requires further studies.

In summary, for the instrument channel segment of the suction-biopsy channel, the sponge ATP test method works well and our the simulated-use data confirmed that when the endoscope manufacturer’s cleaning instructions are followed that the ≤100 RLUs cut-off for this ATP test kit should be reliably achieved. Further studies are warranted to determine how well this 100 RLU cleaning cut-off works in a busy endoscopy clinic.
